# Waist circumference as a mediator of biological maturation effect on the motor coordination in children

**DOI:** 10.1016/j.rppede.2016.02.004

**Published:** 2016

**Authors:** Leonardo G.O. Luz, André Seabra, Cristina Padez, João P. Duarte, Ricardo Rebelo-Gonçalves, João Valente-dos-Santos, Tatiana D.D. Luz, Bruno C.M. Carmo, Manuel Coelho-e-Silva

**Affiliations:** a Laboratório de Cineantropometria, Atividade Física e Promoção da Saúde (Lacaps), Universidade Federal de Alagoas (Ufal), Arapiraca, AL, Brazil; b Centro de Investigação em Atividade Física, Saúde e Lazer (CIAFEL), Universidade do Porto, Porto, Portugal; c Centro de Investigação em Antropologia e Saúde (CIAS), Universidade de Coimbra, Coimbra, Portugal; d Universidade de Coimbra, Coimbra, Portugal; e Universidade Lusofona de Humanidades e Tecnologias, Lisboa, Portugal

**Keywords:** Biological maturation, Physical fitness, Anthropometrics, Children

## Abstract

**Objective:**

The present study aimed to: 1) examine the association of biological maturation effect on performance at a motor coordination battery and 2) to assess whether the association between biological maturation and scores obtained in motor coordination tests is mediated by some anthropometric measurement.

**Methods:**

The convenience sample consisted of 73 male children aged 8 years old. Anthropometric data considered the height, body mass, sitting height, waist circumference, body mass index, fat mass and fat-free mass estimates. Biological maturation was assessed by the percentage of the predicted mature stature. Motor coordination was tested by the Körperkoordinationstest für Kinder. A partial correlation between anthropometric measurements, *z*-score of maturation and the motor coordination tests were performed, controlling for chronological age. Finally, causal mediation analysis was performed.

**Results:**

Height, body mass, waist circumference and fat mass showed a slight to moderate inverse correlation with motor coordination. Biological maturation was significantly associated with the balance test with backward walking (*r*=-0.34). Total mediation of the waist circumference was identified in the association between biological maturation and balance test with backward walking (77%).

**Conclusions:**

We identified an association between biological maturation and KTK test performance in male children and also verified that there is mediation of waist circumference. It is recommended that studies be carried out with female individuals and at other age ranges.

## Resumo

**Objetivo:**

O presente estudo teve como objetivos: 1) analisar a associação do estado maturacional com o desempenho nas provas de coordenação motora em crianças e 2) examinar se a relação entre o estado maturacional e o desempenho no KTK é mediada por alguma medida antropométrica.

**Métodos:**

A amostra de conveniência foi composta por 73 crianças do sexo masculino com 8 anos. A antropometria considerou a estatura, massa corporal, altura sentado, o perímetro de cintura, índice de massa corporal, as estimativas de massa gorda e massa livre de gordura. A maturação biológica foi avaliada pelo percentual da estatura matura predita. A coordenação motora foi testada pela bateria *Körperkoordinationstest für Kinder*. Foi feita a correlação parcial entre as medidas antropométricas, z-escore da maturação e as provas de coordenação motora, com controle para idade cronológica. Por último, a análise de mediação causal foi feita.

**Resultados:**

Estatura, massa corporal, perímetro de cintura e massa gorda apresentaram correlação inversa de magnitude pequena a moderada com as provas de coordenação motora. A maturação biológica associou-se significativamente à prova de equilíbrio em marcha à retaguarda (r=-0,34). Foi identificada mediação total do perímetro de cintura na relação do estado maturacional com o equilíbrio em marcha à retaguarda (77%).

**Conclusões:**

Foi possível identificar a associação entre o estado físico maturacional e o desempenho em uma prova de bateria de coordenação motora em crianças do sexo masculino e, ainda, afirmar que há mediação do perímetro de cintura. Recomenda-se que estudos sejam feitos com indivíduos de outras idades e do sexo feminino.

## Introduction

The motor coordination of children seems to be associated with health-related physical fitness,^1^ physical activity,^2^ body composition,^3^ sociodemographic characteristics,^4^ and consequently the overall health of this population. However, motor development in prepubertal ages appears to influence decisively the motivation^5^ and even the involvement in motor practices in adolescence, such as games and sports.^6^

The Körperkoordinationstest für Kinder (KTK) test has been used to evaluate motor coordination in children and adolescents.^7^^,^ 8 However, there is a lack of studies in the literature describing the multiple factors that may be related to motor development of children, including the biological maturation. The studies performed with the KTK test battery aimed primarily at the association of the anthropometric characteristics of children and youth, especially body mass index (BMI), with test performance.^7^^,^ 8 However, in a recent systematic review with meta-analysis of studies on the subject, no selected study took into account the relationship between maturational status and BMI values of individuals, as well as performance on motor coordination tests.^7^

The maturational status has been related to physical activity^9^ and physical fitness of young subjects.^10^ However, the most popular measures of biological maturation result from sexual maturation stages that are unique to the pubertal years and do not correspond to a continuous scale, likely to be used in a correlational design.^11^ Katzmarzyk et al.^10^ used the skeletal maturation method and the results showed the complexity of the interrelations between body size, sexual maturation, and physical fitness. Still, the authors stated that the effects of biological maturation in children are mainly expressed through body size and that maturational status was the strongest influence on the physical performance of children.

Therefore, the aim of this study was to evaluate the association of maturational status with performance on KTK tests in prepubertal children and the relationship between biological maturation and performance in KTK mediated by some anthropometric measurement of individuals.

## Method

This is a descriptive study in which data were collected at a single time point and represent a cross-section of the characteristics of individuals in the study. The four schools in Arapiraca and Alagoas, Brazil, were selected through non-probability sampling accessibility. The selection criterion was only the schools' stratification according to its public (*n*=2) and private (*n*=2) nature. Informed consent was given to all male children in the age group of interest for the study, as well as to their guardians. The sample consisted of 73 male students, aged between 8 and 8.99 years, representing 90% of the eligible children number. Not giving informed consent, absence on the day of data collection, or physical disability to perform the test battery were exclusion criteria. The study was designed and conducted in compliance with international standards of experimentation involving human subjects (Helsinki Declaration of 1975) and was duly approved by the Institutional Review Board of the Federal University of Alagoas, registered under the Opinion CAAE 09200413.5.0000.5013.

The anthropometric measurements were: height, body mass (BM), sitting height (SH), waist circumference (WC), and skinfolds, all measured at school in the same period of the day. Height (0.1cm) and SH (0.1cm) were measured with a portable stadiometer (Sanny Caprice, São Paulo, Brazil). BM (0.1kg) was measured on a digital scale (Techline, São Paulo, Brazil). The students wore only light clothing and were barefoot, remained with the arms relaxed at their sides. WC (0.1cm) was measured at midpoint between the last rib and iliac crest, at the time of minimum breathing, with an anthropometric steel measuring tape (Sanny Medical Starrett, São Paulo, Brazil). Subscapularis, triceps, and leg skinfolds (1mm) were measured with Lange skinfold caliper (Beta Technology, Santa Cruz, California, USA), from an average of three measurements collected at each preset anatomical point, in a rotational order, on the right side of the subjects. The procedures used were based on the instructions described by Lohman et al.^12^ Body mass index (BMI) and fat percentage^13^ were calculated and used for BM fragmentation in fat mass (FM) and fat-free mass (FFM). Technical error of measurement and reliability coefficient of anthropometric variables were obtained by test-retest, with one week interval in a group of 19 children. The respective values were: height (0.6cm; 0.98) body mass (0.6kg, 0.99), waist circumference (1.6cm; 0.93), sitting height (0.5cm; 0.96), and skinfolds (1.0-1.4mm; 0.94-0.98).

Biological maturation evaluation was performed using the percentage of mature height achieved at a given time. The percentage of the predicted mature height (%PMH) obtained at a certain age with the Khamis and Roche method^14^ is considered as a noninvasive method and provides data in a continuous format. The measurement, as a continuous variable, is moderately associated with bone age, considered a benchmark of biological maturation.^9^^,^ 15 To assess the biological maturation, %PMH was expressed as *Z*-score on the mean and standard deviation for age and sex for the sample of the Berkeley Guidance Study, University of California.^16^ Maturation *Z*-scores are often used to assess biological maturation: average-maturing (*z*-score between -1.0 and 1.0); late-maturing (*Z*-score<-1.0); early-maturing (*Z*-score>1.0.^17^^,^ 18). In the present study, the parents' heights were self-reported and maturation *z*-score was the variable used to represent the biological maturation of individuals.

Motor coordination was evaluated with the body coordination test for children (Körperkoordinationstest für Kinder - KTK).^19^ The choice was based on the positive aspects highlighted by Cools et al.^20^ Psychometric characteristics of KTK^19^ indicate a test-retest reliability coefficient for each test separately, ranging between 0.80 and 0.96. Its application requires a space with an area of 4×5 meters. KTK has in its final form four tests: balance in walking backward (WB), lateral jumps (LJ), lateral transposition (LT) and monopedal jumps (MJ). The subjects were not familiar with the tests prior to testing. In the present study, we considered the isolated performance in each KTK test. Thus, the values standardized by the original authors^19^ were not used nor the motor ratio was calculated, as the original study was intended to obtain a categorical assessment of children and young people with motor deficits. The same procedure was adopted in another study.^21^ This decision was based on: 1) lack of study showing the cross-cultural validity of the score suggested by the original authors of the results of each test in Brazilian children; 2) absence of solid information on the validity of motor ratio cutoff values in Brazilian children; 3) having no knowledge of the clinical and pedagogical significance of the proposed classification by the German authors; 4) the fact that our study sample comprised only individuals of the same sex and same age group; and 5) the fact that the main objective of the study is to examine the association of anthropometric and biological maturation variables with the performance on KTK tests.

The evaluations were conducted at the premises of schools. Evaluations in each school lasted four weeks. Each week, an average quantity of 10 individuals was evaluated. Regarding evaluation times, first anthropometric measurements were taken, and in another week, KTK was applied individually with children wearing shoes. The sequence of KTK's evidence was uniformly applied to subjects in the following order: balance in walking backward, lateral jumps, lateral transposition, and monopedal jumps.

Descriptive statistics of central tendency and dispersion were determined and the normality of the distribution was further tested with the Kolmogorov-Smirnov test. Variables that did not meet the assumptions of normal distribution were subjected to logarithmic transformation for the inferential analysis. However, we chose to present the original values in the tables of results. Subsequently, the partial correlation test was performed, controlled by chronological age, between anthropometric variables (height, body mass, BMI, sitting height, waist circumference, fat mass, and fat-free mass), maturation *Z* score, and results for each KTK test. Correlation coefficients were interpreted according to Hopkins et al.^22^ After correlations, in order to examine how much of the association between biological maturation and performance in KTK was mediated by anthropometric characteristics, linear regression models were adjusted based in the procedures described by Baron and Kenny.^23^ The first equation has the mediator (anthropometry) and the independent variable (biological maturation *Z* score). The second equation uses the dependent variable (KTK score) and the independent variable (biological maturation *Z* score). The third equation assessed the dependent variable (KTK score) with the independent variable (biological maturation *Z* score) and the mediator (anthropometry). The following criteria were used to establish a mediation: 1) the independent variable should be significantly related to the mediator; 2) the independent variable should be significantly related to the dependent variable; 3) the mediator should be significantly related to the dependent variable; and 4) the association between the independent variable and dependent variable should be attenuated when the mediator is included in the regression model. Finally, mediation is tested with the steps described by Sobel^24^: first, an estimated attenuation or indirect effect (i.e., the effect of independent variables on the mediator, equation 1, multiplied by the mediator effect on the dependent variable, equation 3); and, second, the indirect effect is divided by the effect calculated in equation 2. A significance of *p*<0.05 was considered in the analysis. The SPSS 22.0 software (SPSS, Inc., Chicago, IL) was used.

## Results

Descriptive results are shown in Table 1 . Regarding biological maturation, the subjects are in the average percentage of the predicted mature height of 74.7%, with a small standard deviation magnitude (±1.6). The same was not true in KTK tests. The lateral transposition test is the one with less variation in the standard deviation values.

**Table 1:** General characteristics of total sample (*n*=73).

Variables	Range			Mean		Standard deviation
	Minimum	Maximum		Value	(95%CI)	
Chronological age (years)	8.00	8.99		8.52	(8.45-8.59)	0.30
Predict mature height (cm)	161.6	188.7		175.4	(173.9-176.9)	6.4
Predict mature height (%)	72.2	78.6		74.7	(74.4-75.1)	1.6
Height (cm)	119.2	146.2		131.1	(129.8-32.5)	5.8
Body mass (kg)	18.1	61.7		31.4	(29.6-33.3)	7.9
Body mass index (kg·m^-2^)	12.0	30.8		18.1	(17.3-18.9)	3.4
Sitting height (cm)	41.8	79.0		68.3	(67.3-69.4)	4.5
Waist circumference (cm)	48.0	92.8		61.6	(59.7-63.4)	7.9
Fat mass (kg)	1.5	40.1		8.4	(7.0-9.9)	6.2
Fat-free mass (kg)	16.6	30.3		23.0	(22.3-23.6)	2.9
Backward balance^a^	3	68		37.8	(34.4-41.2)	14.4
Lateral jumps^a^	11	57		33.2	(31.0-35.5)	9.7
Lateral transposition^a^	18	60		32.4	(30.8-34.0)	6.8
Monopedal jumps^a^	9	65		36.6	(33.8-39.4)	12.1

a There is no measurement unit.

Table 2 shows the partial correlation coefficients between anthropometric variables, biological maturation (maturation *Z* score), and performance in each KTK test, controlled for the spurious effect of chronological age. Biological maturation did not correlate significantly with most KTK tests, notably lateral jumps, lateral transposition, and monopedal jumps. In contrast, there was a significant inverse correlation of moderate magnitude with the balance in walking backward (*r*=-0.34). Height, body mass, BMI, waist circumference, and fat mass were inversely and significantly associated with maturation *Z* score and balance in walking backward KTK test, ranging between weak and moderate magnitudes.

**Table 2:** Partial correlation coefficients between anthropometric variables and performance on motor coordination tests (KTK), controlled by chronological age.

Anthropometric variables	Maturation *Z* score	KTK			
		Backward balance	Lateral jumps	Lateral transposition	Monopedal jumps
Height	0.52^a^	-0.25^a^	-0.42^a^	-0.26^a^	-0.24a
Body mass	0.68^a^	-0.36^a^	-0.41^a^	-0.27^a^	-0.30^a^
Body mass index	0.60^a^	-0.35^a^	-0.30^a^	-0.22	-0.29^a^
Sitting height	0.42^a^	-0.16	-0.18	-0.24^a^	-0.23
Waist circumference	0.67^a^	-0.44^a^	-0.43^a^	-0.27^a^	-0.36^a^
Fat mass	0.68^a^	-0.37^a^	-0.37^a^	-0.27^a^	-0.33^a^
Fat-free mass	0.40^a^	-0.13	-0.34^a^	-0.27^a^	-0.14
Maturation *Z* score	-	-0.34^a^	-0.17	-0.10	-0.15

a *p*<0.05.

**Figure 1:**
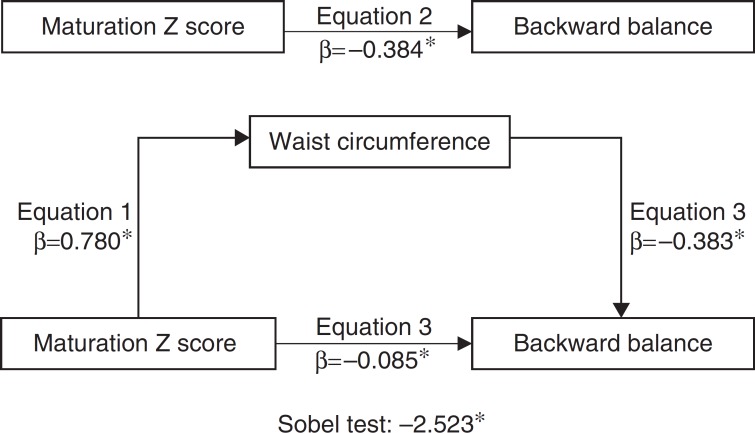
Measurement model of waist circumference on the relationship between biological maturation and performance on backward balance of motor coordination battery, controlled by chronological age (*p<0.05).

Of all anthropometric variables showing significant association with the balance in walking backward KTK test, waist circumference was the only one to present itself as a mediator of the relationship between the biological maturation and test performance (Fig. 1). The results show that the effect of biological maturation on performance in the balance in walking backward KTK test acted as full mediator of waist circumference (77%; *Z*=-2.523, *p*<0.05).

## Discussion

The present study found an inverse association between biological maturation and balance in walking backward KTK test, whose correlation coefficient showed moderate magnitude. Waist circumference was the only anthropometric variable acting as mediator in the relationship between biological maturation and KTK test performance, notably in the balance in walking backward test.

Biological maturation has been taken into account in studies involving the pediatric population. Currently, the relationship between biological maturation and level of physical activity^9^ and motor performance^10^ are issues reported in the literature. However, it is noteworthy that, normally, the studies consider "motor performance" as performance in physical fitness tests related to health, and not necessarily in motor coordination tests. Few studies with children have considered both biological maturation and performance on tests of motor coordination or motor skills.^25^^,^ 26 Recently, Freitas et al.^25^ assessed the contribution of skeletal maturation in the performance of KTK tests in children aged 7-10 years. The authors stated that, in most cases, the correlation coefficients are negative, suggesting that a later biological maturation is associated with better results in test trials. Also, they concluded that biological maturation alone, or combined with body size, has little influence on KTK results.

In a longitudinal study, Deus et al.^27^ followed the performance of children aged 6-10 years in KTK tests and the results showed that the coordinative competency of KTK tests showed distinct trajectories. The balance in walking backward test was the only one that showed a linear trajectory, which did not occur in other tests, and demonstrated in its results that the higher the starting value, the lower the annual gains (*r*=-0.55). Moreover, the findings of Deus et al.^27^ also showed that BMI is an essential factor for the good performance of this test. In fact, this test requires the displacement of the center of gravity in a balanced manner, which may penalize children with higher body fat, mainly located in the trunk area. D'Hondt et al.^28^ also reported that there is an inverse relationship between body fat and performance on KTK tests, which seems more pronounced in those with more advanced age. A possible explanation for these findings in the literature may be that motor development of children increases as they become more mature. However, the more advanced the biological maturation of an individual tends to be, this advancement tends to be slower and stabilize.^11^ On the other hand, weight gain that is also related to biological maturation, tends to increase, which would contribute to a greater chance of having an inversely proportional association between body mass and performance on KTK in children with advanced biological maturation.

In recent study with children of both sexes, Lopes et al.^8^ reported that, in addition to BMI, waist circumference, height-waist ratio, and body fat percentage also were associated with performance on KTK. However, fat percentage (*β*=2.395, 95%CI 1.234-4.646; *p*=0.010) showed higher sensitivity to predict low motor coordination in girls. For males, increased value of waist circumference (*β*=3.296, 95%CI 1.784-6.090; *p*<0.001) was more prevalent in the association with low performance on KTK. Such findings are in line with the results of our study, as it was found inverse relationship between biological maturation and performance on the balance in walking backward test mediated by central fat, characterized by the waist circumference values.

Given the above, our findings show evidence that biological maturation, although in small proportion, is correlated with the performance on KTK in prepubertal male children, especially by the inverse correlation of moderate magnitude obtained on the balance in walking backward test. However, one cannot conclude the same for other KTK tests. These findings support those in the literature and raise evidence that the development of children coordination is not only related to the influence of biological maturation, but also to the behavioral and environmental influences and their interactions.^25^^,^ 29 ^,^ 30

The present study is one of the few that take into account the children biological maturation in relation to their performance on motor coordination test. However, some limitations should be recognized. Given that we used a cross-sectional design of data collection and the sample composition was carried out in a non-random manner with only males of a single region of the State of Alagoas and without sample size calculation, it is not recommended to generalize the results to other children who do not meet the sample characteristics of the study. Two other aspects to be mentioned are related to the fact that there was no measurement of the variable level of physical activity in children for control purposes and the fact that parental heights were not directly measured for %PMH calculation. However, the results contribute to the knowledge of the coordination performance of children. Furthermore, it denotes the possibility of interference of biological maturation in the relationship of body size and the results of motor coordination, especially on KTK test battery.

In conclusion, taking into consideration that the motor coordination development is of paramount importance in childhood, for its characteristic of predicting physical activity in subsequent stages of life,^6^ it is evident that the results reinforce not only the need for attention to the knowledge of biological maturation of individuals, but mainly highlights the concept that growth, maturation, and motor development are biocultural phenomena.^11^ Future studies with individuals of other age groups are recommended, with larger sample and assessing the effect of maturation on performance with other methods of motor coordination assessment in the pediatric population.
